# Creating healthy eating and active environments in early learning settings: protocol of the CHEERS eHealth intervention study

**DOI:** 10.3389/fnut.2024.1337873

**Published:** 2024-02-28

**Authors:** Lynne M. Z. Lafave, Joyce Hayek, Alexis D. Webster, Ceilidh McConnell

**Affiliations:** CHEERS Lab, Department Health and Physical Education, Mount Royal University, Calgary, AB, Canada

**Keywords:** early childhood education and care, nutrition, physical activity, online intervention, eHealth, educators, Health Promotion

## Abstract

**Background:**

Early childhood educators through their daily interactions with children, play a central role in shaping young children’s health behaviors. Given their influential role, early childhood educators are often targeted in interventions aiming at enhancing their nutrition and physical activity practices.

**Methods:**

This paper presents the design of the CHEERS eHealth program to improve nutrition and physical activity practices within Early Childhood Education and Care (ECEC) centers. The study has a longitudinal quasi-experimental design with recruitment of ECECs across Alberta Canada. ECEC intervention group educators complete 12 weekly online nutrition and physical activity modules and participate in weekly communities of practice sessions to discuss practical applications within their centers. Outcome assessments are scheduled at baseline (T1), mid-point at 5 months (T2), and end of program after 10 months (T3). Outcome measures include the Creating Healthy Eating and Active Environments survey (CHEERS), Mindful Eating Questionnaire (MEQ), Canadian Behavior, Attitude and Nutrition Knowledge Survey (C-BANKS 2.0), Physical Literacy Knowledge, Attitude, Self-Efficacy, and Behavior (PLKASB-ECE), the Environment and Policy Assessment and Observation (EPAO) derived variables, and an objective measure of children’s physical activity using ActiGraph GT3X accelerometers. Linear mixed model analyses will be used to evaluate the effectiveness of the intervention. Qualitative assessments comprise exit interviews and open-response questions embedded within the educational modules.

**Results:**

Preliminary baseline data from the 2019 cohort indicate no statistically significant differences between the intervention and control groups for the primary outcome variables, except age. Educators’ personal nutrition-related knowledge, attitude and behaviors were positively associated with their self-assessments of the nutrition environment and practices in ECECs. A significant correlation was observed between educators’ self-reported physical activity practices and observed activity practices. The CHEERS survey Food Served subscale showed a positive correlation with the objective measures of EPAO-Foods Provided and Nutrition Policy subdomains.

**Discussion:**

We propose that this eHealth intervention would be an effective scaling up approach to enhancing the nutrition and physical activity environments of ECECs by fostering improved nutrition and physical activity-related knowledge, attitudes, and adherence to best practices which will potentially lead to improved outcomes for children in their care.

## 1 Introduction

Early childhood experiences and the environment in which they occur, can shape and influence cognitive and behavioral outcomes in later life (1). Research supports the strong link between early childhood development and adult health (2). Lifestyle habits such as eating and physical activity habits are examples of behaviors that take root during early life stages and persist into adulthood (3, 4). Poor dietary patterns and sedentary behavior during childhood are associated with increased risk of adult obesity, type II diabetes, cardiovascular disease, dyslipidemia and hypertension (5–8). On the other hand, healthy behaviors have been shown to have protective effects (9–11). What is more, good health in early childhood positively impacts cognitive functioning and learning capacities, setting the stage for academic success and better future prospects (12, 13). The period of early childhood is thus a critical time to build foundations for healthy behaviors.

In Canada, research shows that the diet quality of children and youth is poor with suboptimal fruit and vegetable intake (14, 15). In addition, 13.8% of total energy consumption comes from free sugar including added sugars and sugars naturally present in honey, syrups, fruits juices and concentrates (16), exceeding the World Health Organization’s recommended limit of 10% (17). Children’s behaviors, including health behaviors, have been hypothesized to be influenced by layers of their environment (18). The microenvironment is the layer closest to the child and has the strongest and earliest influence on a child’s development and behaviors (18). Early learning settings are part of this microenvironment and are considered among the first and primary settings in which health behaviors are developed and nurtured (19). These settings play a significant role in shaping children’s lifelong habits related to eating and physical activity (20). Participation in early learning and childcare in Canada has witnessed a surge over the past two decades with approximately 60% of children enrolled in some form of early childhood education (21). Children attending full-time day care centers can spend up to 6 h or more per day in these facilities (22) and are consequently assumed to get a significant portion of their nutritional needs in those centers (23). Hence, early childhood settings are critical venues for fostering healthy eating and activity behaviors of young children in Canada.

Early childhood educators are an important part of early learning settings and play a major role in shaping children’s health behaviors (24). Early childhood educators can influence children’s eating and activity behaviors in many ways. They serve as role models, control access to food, provide opportunities for movement, and support children’s self-regulation skills (24–27). Early childhood educators’ personal knowledge and beliefs with regards to nutrition has been found to impact their feeding practices within care settings (28). Misconceptions and inaccuracies in personal knowledge and a lack of training can negatively impact the child care nutrition environment (28–30). On the other hand, research has shown that training opportunities for educators on evidence-based guidelines has the potential to enhance their practices leading to improved health outcomes for children (31–33). Health interventions targeting nutrition and physical activity in early learning settings are increasingly acknowledged for their effectiveness in promoting healthy behaviors early in life (34) aligning with the objectives of Sustainable Development Goal 3 which aims to ensure health and wellbeing for all (35).

The high level of reach and availability of the internet has made it a promising channel to scale up and deliver behavioral change health interventions (36–38). Online or eHealth interventions have gained traction as they enable participant engagement anytime-anywhere, provide repetitive access opportunities, and a wider reach at a lower cost (39–41). Web-based interventions in Early Childhood Education and Care (ECEC) settings have been shown to be highly acceptable and effective in improving nutrition knowledge and practices (42, 43). Within the Canadian context, eHealth interventions in ECEC settings remain limited (44, 45), particularly those designed to support child care educators align with best practice nutrition and physical activity guidelines. Given the available evidence supporting the need of a comprehensive intervention within the ECEC setting along with the effectiveness and sustainability of online modalities, the purpose of this paper is to describe the design of a virtual eHealth nutrition and physical activity intervention aimed at improving the knowledge, beliefs and adherence to best practices of early childhood educators in Alberta, Canada.

## 2 Materials and methods

### 2.1 Study design

The “*CHEERS HEAPful of FUN: raising healthy Albertans*” (CHEERS program) is a health and wellness virtual educational support program that aims to improve the knowledge and skills of early childhood educators and support the implementation of evidence-informed best nutrition and physical activity practices within ECEC settings. The study follows a quasi-experimental design and uses the Social Cognitive Theory for behavior change as a foundation (46). The Social Cognitive Model posits the mutual influence and interplay between behaviors, personal factors and the environment, also known as “*Reciprocal Determinism*” (46, 47). Health behaviors are influenced by personal factors and the physical and social environment, and in turn, individuals can influence their environment through behavior (46). This study integrates key constructs of the Social Cognitive Theory and addresses within-person influences such as educators’ knowledge and beliefs to encourage the adoption of best nutrition and activity practices which in turn can promote healthier ECEC environments. The Social Cognitive Theory was used as a guiding model for the development of the CHEERS materials. The modules were designed to improve educators’ skills and knowledge, aligning with the Social Cognitive Theory constructs, and provide essential information, empowering educators to implement best practices. Another key application of the Social Cognitive Theory pertains to the construct of self-efficacy. Within the Communities of Practice sessions that follow the modules, educators engage in a social negotiation to interpret and integrate the knowledge gained from the modules within their work as early childhood educators to reshape their efficacy beliefs of implementing best practices.

### 2.2 Participants and recruitment

#### 2.2.1 Childcare centers and educators

Participant recruitment was implemented in the province of Alberta, Canada between 2019 and 2022. Children’s Services, Early Childhood Development Branch from the Alberta government, provided the research team with the full list of ECEC programs. ECEC centers were randomly selected for recruitment using postal codes to stratify the selection of ECEC centers from large urban population centers (population >100,000), medium population centers (30,000–99,000), small population centers (1000–29,999), and rural areas (population <1000) throughout the province (48). Starting in 2019, center directors were invited to participate through phone correspondence and provided with general information on the study. Center directors had the option to be in the intervention or control group based on their assessment of both available time and capacity to participate. Centers that agree to participate receive an email package with instructions, a consent form, links to surveys, and contact information of a trained research associate to answer potential questions. Inclusion criteria include: (1) licensed facility-based centers (2) providing care for a minimum of 15 children aged 2–5 years, (3) access to a computer and internet connection and (4) not currently enrolled in any other intervention to improve nutrition and activity practices. Exclusion criteria include (1) unlicensed ECEC centers, and (2) family day home or after school care program. Each participating center was requested to identify a minimum of three staff members to be included in the study, with a preference to include two educators and the center director or manager.

#### 2.2.2 Children

Children were recruited to objectively measure physical activity and sedentary levels from participating ECEC centers. Children were recruited from the classroom of participating educators consisting of children aged between 2 and 5 years. Inclusion criteria include: (1) prior written consent from a parent or guardian to participate; (2) be aged between 2 to 5 years; and (3) be enrolled full-time at the center. Center staff were asked to distribute informational flyers and consent forms to parents via center communication methods with parents. Flyers provided parents with information about the study, what children would be doing as part of the study, and phone numbers of research staff. Parents/guardians provided written consent and answered demographic questions related to birth date and sex when returning the consent forms. Completed consent forms were collected by research staff for child participation and children provided verbal assent during data collection.

### 2.3 Development of materials

The online educational modules were developed in 2019 by a team of content expert developers in nutrition (early childhood dietitians in provincial health authority), physical activity (Be Fit For Life Center), wellness (university faculty member wellness specialist) and early childhood (university faculty member in early childhood) and used evidence-informed best practices to ensure high-quality content. These modules were then sent for review by content expert reviewers, identified from each of the specialties, and revised as necessary prior to implementation. Modules were further reviewed by an Early Childhood Educator university faculty member to ensure the messaging was respectfully communicated.

### 2.4 Intervention procedure

The CHEERS program is a quasi-experimental study with repeated measures that started in 2019 and will continue until 2023. Each year the program is repeated and involves a new cohort with repeated measurements at baseline, 5 and 10 months from baseline. During this period, ECEC centers in the intervention group participate in a series of activities. Firstly, they complete a set of online surveys hosted on Qualtrics. In addition to the surveys, the participants in the intervention group complete a 12-week set of online professional development modules and attend virtual weekly Communities of Practice sessions. The 5-month measurement served as a mid-point assessment and timed after the completion of the online modules and online Communities of Practice sessions. The 10-month measurement, a post intervention follow-up, aimed to evaluate the sustained and long-term impact of the intervention.

#### 2.4.1 Online modules

The modules cover topics on healthy eating patterns and the eating environment, physical activity and literacy, self-care for educators, parents as partners, and the importance of sleep for children. The full list of modules can be found in Table 1. The modules are hosted on Articulate 360 (Articulate Global Inc) with the content authoring tool Rise, which offers several embedding features and pre-built interactions such as card sorting, flashcards, knowledge checks, and click-through processes. The modules include clear learning objectives, instructional videos, links to useful resources and embedded reflection questions that can be accessed through computer or mobile devices. Participants in the intervention group are asked to complete one module per week, with each module taking 1 to 2 h to complete. An example module can be seen in the Supplementary material. Each module requires participants to submit their name and email address as an exit response, which is used to track module completion. This allows the research team to send a reminder email to only those participants who have not yet completed the material. This approach ensures participants have the opportunity to complete the modules before moving to the next phase of the program.

**TABLE 1:** Module topics.

Module 1: Physical literacy–activity for health	Module 7: Nutrition–mealtime experiences
Module 2: Nutrition–what is on the plate?	Module 8: Physical literacy–free play
Module 3: Self-care: you first!	Module 9: Physical literacy– environments matter
Module 4: Nutrition–the feeding relationship	Module 10: Nutrition–curriculum connections
Module 5: Self-care–managing stress	Module 11: Parents as partners
Module 6: Self-care strategies	Module 12: Sleep and health

#### 2.4.2 Communities of practice

Participants engage in two different Communities of Practice throughout the intervention. First while completing the weekly educational modules participants attend online Communities of Practice which provide opportunities to reflect on the practical applications of the module topics with other directors and educators participating in the program. Second, after completing the 12-week modules, participants attend online Communities of Practice meetings with the other participants located at their center. These meetings are facilitated by Health Promotion Leaders who are experts in nutrition, public health, or physical activity. During the post-educational Communities of Practice meetings participants: co-collaborate on strategies to integrate the learning into programming within their respective centers; share challenges they are facing and strategies to overcome them; and are provided with additional resources from the Health Promotion Leaders as needed to support change within their centers. These Communities of Practice meetings take place over a period of 5 months with a session scheduled each month.

#### 2.4.3 CHEERS challenge

Finally, participants are invited to take part in the “CHEERS Challenge” where they are encouraged to utilize the resources gained through the program and mobilize the knowledge they learned to other staff in the center or with parents.

#### 2.4.4 Incentives

Participants in the control group receive an honorarium of $25 for completing surveys at baseline and then again at 10 months. Intervention participants are compensated for their time in the program with a knowledge grant during the 12-week education modules ($300) and the after 5-month Health Promotion Leader communities of practice component ($300). Participants are invited to a wrap up session at the end of the program to share preliminary study findings and celebrate the community.

#### 2.4.5 Control group

Participants in the control group are instructed to continue with their usual practice, with the control group only filling surveys at baseline and end of program with no access to the intervention components.

### 2.5 Data collection and outcome measures

This study follows a mixed-method approach to evaluate the impact of the program in changing practices at the level of ECEC centers. Questionnaires are administered online using Qualtrics platform that is compatible across mobiles and computers. Surveys are administered at three time points: baseline (T1), after 5 months (T2), and after 10 months at the end of the program (T3). Outcome measures are summarized in Table 2.

**TABLE 2:** List of outcome measures.

	Method	Baseline (T1)		Mid-point (T2: 5 months from baseline)		End (T3: 10 months from baseline)	
		**IV**	**CTRL**	**IV**	**CTRL**	**IV**	**CTRL**
**ECEC measures**							
CHEERS (self-reported assessment of ECEC nutrition and physical activity environment)	Online	X	X	X		X	X
PLKASB-ECE (self-reported measure of educators’ knowledge, attitude, self-efficacy and behaviors related to physical literacy in ECECs environment)	Online	X	X	X		X	X
EPAO (observational tool of nutrition and physical activity environments in ECECs)	Observational	X	X			X	X
**Educator measures**							
MEQ (self-reported mindful eating behaviors of educators)	Online	X	X	X		X	X
C-BANKS 2.0 (self-reported dietary knowledge, attitude and behaviors of educators)	Online	X	X			X	X
Self-reflection questions	Online	X					
Exit interviews	Online					X	
**Child measure**							
Physical activity	ActiGraph Accelerometer	X	X			X	X

IV, intervention; CTRL, control.

#### 2.5.1 Quantitative assessments

##### 2.5.1.1 CHEERS

The CHEERS survey is a community-based educator-administered self-audit survey designed to offer ECEC centers an assessment of their center’s healthy eating and physical activity environment (49). Each of the center’s participating educators complete the CHEERS tool which includes 59 items and measures four constructs: Food Served- *n* = 23 items, (e.g., My childcare center serves vegetables and fruit prepared with little or no added fat, sugar or salt), Healthy Eating Environment- *n* = 18 items, (e.g., My childcare center provides children with an assigned area, with few distractions, to sit and eat), Healthy Eating Program Planning- *n* = 6 items, (e.g., My childcare staff members attend professional development on nutrition education), and Physical Activity Environment- *n* = 12 items, (e.g., My childcare center has indoor space available for physical activity). Items are measured using a seven-point scale with response options ranging from 1 = “never” to 7 = “always.” The four subscale scores are calculated using an average of the items in the grouping. The CHEERS score is calculated as the average of the four subscales with higher scores reflecting adherence with optimal practices in nutrition and physical activity. The CHEERS survey provides ECEC centers with an assessment of their eating and activity environments and provides a personalized report that identifies one area of strength and one area for improvement in each of the four measured domains. The development of the CHEERS tool followed a structured content validation process with a final Flesch–Kincaid readability grade 8.1 (50). It has also been assessed within the educator community and shows evidence of good internal consistency (Cronbach’s α = 0.91), intra-rater reliability (ICC = 0.81), inter-rater reliability (ICC = 0.59), and concurrent validity with direct observation (ICC = 0.65) (51) as well as online administration (ICC = 0.86) (52).

##### 2.5.1.2 Mindful Eating Questionnaire (MEQ)

The Mindful Eating Questionnaire is a 28-item self-reported scale that measures mindful eating behaviors (53). Each of the center’s participating educators complete the MEQ measures which include five domains: disinhibition, awareness, external cues, emotional response and distraction. It uses a four-point Likert scale with responses ranging from 1 (*never/rarely*) to 4 (*always/usually*), where higher scores reflect greater mindful eating. Each subscale score is calculated as the means of items, excluding the “not-applicable” responses. The total score is calculated as the mean of the five subscales with a higher score reflecting more mindful eating.

##### 2.5.1.3 Canadian Behavior, Attitude and Nutrition Knowledge Survey (C-BANKS 2.0)

The C-BANKS 2.0 is a survey designed to measure nutrition-related knowledge, attitude, and behavior in the Canadian population (54). Each of the center’s participating educators complete the C-BANKS 2.0 which is comprised of 60 items and measures three constructs: Knowledge (20 items), Attitude (5 items) and Behavior (35 items). The C-BANKS 2.0 has been assessed for reliability and validity in Canadian adults (54). Knowledge items are scored as correct (1) or incorrect (0) and summed to create a total score. Attitude items use a seven-point Likert scale ranging from 1 (*strongly disagree*) to 7 (*strongly agree*) and a total attitude score is calculated as the mean of items where a higher score reflects more positive attitudes. Behavior items employ a seven-point Likert scale ranging from 1 (*never*) to 7 (*always*) and the total score is calculated using the average of the items. The total C-BANKS 2.0 score is calculated as the average of the three subscales.

##### 2.5.1.4 Physical Literacy Knowledge, Attitude, Self-efficacy, and Behavior (PLKASB-ECE) questionnaire for early childhood educators

Physical literacy is the motivation, confidence, physical competence, and knowledge and understanding to enable all individuals to value and take responsibility to engage in physical activities for life (55). The PLKASB-ECE is a self-administered instrument that measures early childhood educators’ knowledge, attitude, self-efficacy, and behaviors related to physical literacy in childcare environments. Each of the center’s participating educators complete the PLKASB-ECE which consists of 6 subscales: Knowledge (*n* = 7) measuring educators’ physical literacy knowledge; Perception of Knowledge (*n* = 2) measuring educators’ perception of their understanding related to physical literacy concepts, Self-Efficacy (*n* = 3) measuring educator’s self-efficacy toward developing physical literacy skills in the early learning environment, Attitude (*n* = 3) measuring educators’ attitudes regarding physical literacy and health outcomes; Environment Behaviors (*n* = 3) measuring educators’ perception of their physical literacy promotion practices in the ECE environment; and Personal Behaviors (*n* = 3) measuring perception of personal physical activity behaviors. Knowledge items are scored correct (1) or incorrect (0) and summed to derive the total score with a higher score indicating higher knowledge of physical literacy concepts. All perception, self-efficacy, attitudinal, and behavior items are scored on a seven-point Likert scale from 1 (*strongly disagree*) to 7 (*strongly agree*) and averaged into a mean score for each subscale (56).

##### 2.5.1.5 Environment and Policy Assessment and Observation- EPAO

The EPAO instrument is used to objectively assess the nutrition and physical activity environment of participating ECEC centers. A single EPAO assessment is completed per center which consists of a day-long observation of practices within childcare facilities and includes a document review of the centers’ policies by a member of the research team (57). Trained Health Promotion Leaders (HPLs) visit consenting centers twice per year (Fall and Spring of every year) to complete the EPAO on a day agreed upon between directors and the HPL conducting the observation. Observations of nutrition and physical activity (PA) practices typically take place in the participating educators’ classroom, consisting of children ages 2–5 years. The nutrition related sections of the EPAO assess the overall nutrition environment and compliance with best nutrition practices. This study utilized the following EPAO nutrition-derived variables: Foods Provided, Feeding Environment, Feeding Practices and Nutrition Policy. The physical activity portion of the EPAO measures the following subdomains: outdoor and indoor play, educator’s physical activity practices and professional development, screen time and physical activity policy. In this study, the EPAO-overall PA score was used and calculated by summing all subdomains.

##### 2.5.1.6 Accelerometers

ActiGraph GT3X accelerometers are used to objectively measure physical activity and sedentary levels of a subset of children from the participating ECEC centers. ActiGraph accelerometers have consistently been shown to provide reliable and valid estimates of physical activity in preschool children (58, 59). Educators are trained how to correctly attach and remove accelerometers by research staff. Children wear the accelerometers on the right hip using an elastic waist belt. Prior to placing the accelerometers, children’s assent is also obtained. Children wear the accelerometers for 7 days while they are at the ECEC centers. Children are fitted when they arrived at the child care center and accelerometers are removed at the end of each day. In this study, activity counts are set to 15-s Epochs. A minimum wear-time of 250 min/day for 4 days is required for a valid day. ECEC educators are trained to take children’s weight and height and record all wear-time related information in a daily log. Height is measured to the nearest 0.1 cm using a portable stadiometer (Seca 213 stadiometer) and weight is measured to the nearest 0.1 kg using an electronic scale. Using Pate et al. (60) proposed cut-off points, PA intensity is categorized into: sedentary PA: <200 counts/15 s, light PA: 200-419 counts/15 s, moderate PA: ≥420 counts/15 s and vigorous PA: ≥842 counts/15 s.

#### 2.5.2 Qualitative assessments

##### 2.5.2.1 Exit interviews

Following the completion of the 10-month intervention, educators and directors from the intervention group are invited to have 30-min semi-structured interviews conducted by HPLs via Google Meet. The interviews aim to assess participants’ experiences with the online modules and community sessions, barriers encountered, potential areas for improvement, and changes that occurred in their centers since joining the CHEERS program. The interviews are digitally recorded and subsequently transcribed into written form for further analysis.

##### 2.5.2.2 Modules reflective questions

The 12 online modules contain open-response questions at the end of each educational module. The questions are completed within the SurveyMonkey platform and are intended to stimulate participants’ reflective practice and enhance their overall learning experience.

### 2.6 Statistical analysis

#### 2.6.1 Intervention evaluation

We hypothesize that the CHEERS program will be effective in promoting positive changes in educator’s knowledge, beliefs, and practices leading to significant improvements in ECEC center nutrition and physical activity environments. Linear mixed model (LMM) analyses will be used to analyze the intervention effects on educator’s outcome measures. A three-level model with repeated measurements as first level, educators as second level and ECECs as third level will be used.

A qualitative thematic analysis using the NVivo 10 program will be conducted to explore participants’ perspective, satisfaction and evaluation of the CHEERS intervention, their use of online modules, their experiences, changes in knowledge, attitude, food provision and practices and barriers and enablers influencing adherence to best nutrition and physical activity practices.

#### 2.6.2 Baseline descriptive characteristic and associations

In the current paper, we present baseline characteristics for the intervention and control groups. Additionally, we explore correlations between the CHEERS self-assessment survey and corresponding EPAO subdomains at baseline. We also assess the association between early childhood educator’s baseline personal nutrition knowledge, beliefs, and behavior with the corresponding CHEERS nutrition composite. All analyses are done using SPSS, version 28 (SPSS Inc., Chicago, Illinois) and statistical significance was set at a *p*-value < 0.05. A descriptive analysis was performed using means and standard deviations for continuous variables, whereas numbers and percentages were used for categorical variables. *T*-tests or chi-square tests were conducted to compare baseline demographic characteristics between the intervention and control groups. Fisher’s exact test was also used when the expected frequency was less than 5. Bivariate analyses examining the association between CHEERS and the C-BANKS 2.0 and EPAO subdomains were carried out using Pearson or Spearman correlation tests for non-normal data.

## 3 Results

### 3.1 Demographic profile by group

In 2019, 144 centers were invited to participate, of those 83 center directors (58%) agreed to allow recruitment of educators in their centers. Once the intervention started, five centers withdrew from the study due to three main reasons, change in center director, closure of center, and center staff left the profession resulting in 78 centers total. The intervention group comprised 138 participants (50 centers) and the control group 70 (28 centers). The baseline assessments of the first year (2019) revealed a study population 96.2% female with an average age of 40.05 ± 11.67 years. The majority were educators making up 62% of participants followed by directors accounting for 30.3% of the sample, 1.4% were cooks and 6.3% owners. Most participants were Level 3 which represents an educational completion of a 2-year diploma or university degree (44.4%) while 15.9% were Level 1 which represents the completion of the minimum requirement of the Child Care orientation program. The majority of the ECEC programs were private (70.7%) and provided food (91.4%). At baseline, with the exception of participant age, there were no significant differences in demographic characteristics between intervention and control groups (Table 3).

**TABLE 3:** Characteristics of ECECs and ECEC educators.

Characteristics	All N (%) (*n* = 208)	IV (*n* = 138)	CTRL (*n* = 70)	*p*-value
**Number of children enrolled per center**				
Mean (± SD)	46.17 ± 34.40	47.40 ± 37.16	44.17 ± 29.93	0.694^a^
**Auspice**				
Non-profit	61 (29.3)	40 (29)	21 (30)	0.879^b^
For-profit	147 (70.7)	98 (71)	49 (70)	
**Food provision**				
Center provides food	191 (91.4)	124 (89.2)	67 (95.7)	0.114^b^
Parent provides food	18 (8.6)	15 (10.8)	3 (4.3)	
**Education level of ECEC educators**				
Level 1 (orientation course)	33 (15.9)	23 (16.7)	10 (14.5)	0.340^b^
Level 2 (1-year certificate)	28 (13.5)	18 (13)	10 (14.5)	
Level 3 (2-year diploma)	92 (44.4)	66 (47.8)	26 (37.7)	
University degree	54 (26.1)	31 (22.5)	23 (33.3)	
**Age of ECEC educators**	40.05 ± 11.67	41.49 ± 11.85	37.20 ± 10.82	0.012^a^
**Gender**				
Male	6 (3.8)	6 (5.8)	0 (0)	0.097^c^
Female	151 (96.2)	98 (94.2)	53 (100)	
**Position**				
Director	63 (30.3)	39 (28.3)	24 (34.3)	0.753^c^
Educator	129 (62)	89 (64.5)	40 (57.1)	
Cook/chef	3 (1.4)	2 (1.4)	1 (1.4)	
Owner/operator	13 (6.3)	8 (5.8)	5 (7.1)	

^a^*p*-value for the Independent Samples *T*-test.

^b^*p*-value for the chi-square test.

^c^*p*-value for Fisher’s exact test.

SD, standard deviation; N, sample size; IV, intervention; CTRL, control.

### 3.2 Association of educators’ personal nutrition-related knowledge, attitude, and behavior (C-BANKS 2.0 scores) with CHEERS nutrition subdomains

As shown in Table 4, C-BANKS 2.0 and CHEERS total scores were significantly correlated, *r* = 0.156, *p* = 0.049. Specifically, the dietary behavior subscale of the C-BANKS 2.0 was significantly correlated with all of CHEERS nutrition subdomains (Food Served, Healthy Eating Environment and Healthy Program Planning) (*p* < 0.005). In other words, educators’ personal dietary behavior was significantly correlated with the nutrition environment and practices in the centers. Additionally, educators’ nutrition knowledge and attitudes were significantly positively correlated with their dietary behaviors. Educators with greater knowledge and a more positive attitude had significantly healthier dietary behaviors (*p* < 0.005).

**TABLE 4:** Spearman Correlations between CHEERS and C-BANKS 2.0 for the total sample (*N* = 160) [Adapted from Lafave et al. (54)].

Correlations							
					**C-BANKS 2.0**		
	**CHEERS–NS**	**FS**	**HEE**	**HEPP**	**Knowledge**	**Attitude**	**Behavior**
C-BANKS	0.156*						
Knowledge	0.031	0.029	0.017	0.005	–		
Attitude	0.007	0.050	0.037	0.041	0.232*	–	
Behavior	0.428*	0.348*	0.412*	0.383*	0.256*	0.294*	–

*Correlation is significant at the 0.05 level (2-tailed).

CHEERS NS, CHEERS nutrition score; FS, food served; HEE, healthy eating environment; HEPP, healthy eating program planning.

### 3.3 Association of CHEERS components with corresponding EPAO subdomains

The Physical Activity Environment (PAE) subscale of CHEERS was significantly correlated with the overall EPAO-PA score, *r* = 0.462, *p* = 0.035 (Table 5). This means, educators’ self-assessment of their center’s physical activity as measured by the CHEERS survey was significantly correlated with the physical activity environment and practices as objectively observed using the EPAO. The Food Served (FS) subscale of CHEERS was significantly correlated with the EPAO-Foods Provided subdomain, *r* = 0.549, *p* = 0.010, and with the EPAO-Nutrition Policy subdomain, *r* = 0.438, *p* = 0.047. In other words, the EPAO’s objective measurements of the food provided and the centers’ nutrition policy were significantly correlated with educators’ self-reported scores on food served. No significant associations were found between the self-reported Healthy Eating Environment scores from the CHEERS survey and the EPAO’s objective assessments of feeding environment and practices (*p* > 0.005).

**TABLE 5:** Correlations between CHEERS and EPAO subdomains (*N* = 21).

Correlations				
**CHEERS**				
**EPAO**	**FS**	**HEE**	**HEPP**	**PAE**
Foods provided	0.549^a^*	–	–	–
Feeding environment	–	0.283^a^	0.283^a^	–
Feeding practices	–	0.035^a^	0.19^a^	–
Nutrition policy	0.438^b^*	0.186^b^	0.329^b^	–
Physical activity score	–	–	–	0.462^b^*

^a^Coefficient for the Pearson correlation.

^b^Coefficient for the Spearman correlation.

*Correlation is significant at the 0.05 level (2-tailed).

FS, food served; HEE, healthy eating environment; HEPP, healthy eating program planning; PAE, physical activity environment.

## 4 Discussion

This article describes the protocol of the CHEERS eHealth intervention program which aims to encourage environmental changes of early learning settings at a wide geographical scale (provincial scope). We propose that the intervention would be an effective scaling up public health innovation due to its unique approach of utilizing eHealth strategies to improve the nutrition and physical activity environments of ECECs through staff education and community building to increase knowledge, attitudes, and adherence to best practices. This paper also provides baseline characteristics of the participants who took part in the CHEERS program in the first year and compares self-reported and observed eating and activity practices. In addition, the relation of educators’ own nutrition knowledge, attitudes and behaviors with self-reported evaluations of nutrition environments is examined.

Findings of the current study add to the existing research demonstrating the influence of educators’ own nutrition behaviors on food practices at the ECEC level (61). Our results indicate that educators with higher nutrition knowledge and positive attitudes toward healthy eating were more likely to engage in personal healthy dietary behaviors. Furthermore, the healthy dietary behavior of educators was positively correlated with better nutrition practices at the ECEC level. Educators who adopt healthier eating habits were more likely to provide supportive food environments and feeding practices to children in their care. Our finding that educators’ own dietary behaviors are associated with better mealtime practices and environment is supported by several theories including the Social Cognitive Theory (46) and the Ecological Model (18). Those theories underline the influence of important caregivers’ behaviors on promoting a child’s healthy habits through mechanisms such as normative practices, social support and role modeling. We did not find direct significant correlations between nutrition knowledge and attitudes with the feeding environment. However, our results suggest that educators’ knowledge and nutrition beliefs may influence personal dietary practices which can in turn contribute to the creation of better food environments and feeding practices at the daycare level. Future research should explore the indirect association between early childhood educator’s own nutrition knowledge and attitudes with feeding practices by investigating the potential mediating effect of personal dietary behaviors.

Objective measurement of the physical activity environment as measured by the EPAO-PA subscale was significantly associated with self-reports of physical activity environment and practices. The Physical Activity Environment subscale of the CHEERS survey measures a similar concept to the EPAO, in that they both evaluate daily physical activity opportunities, play equipment use, staff practices, professional development, and physical activity policy. The strong positive correlation between the CHEERS Physical Activity Environment subscale with the EPAO-PA subdomain suggests that the CHEERS self-report measure is closely aligned with the observational measure and provides further evidence of concurrent validity for the CHEERS survey assessing physical activity practices in child care centers (62, 63).

Similarly, the results of our study reveal a strong positive correlation between the Food Served subscale of the CHEERS and the objectively measured EPAO-Foods Provided and Nutrition Policy subdomains. The latter can be explained by the two tools measuring similar constructs. For instance, the Food Served subscale of the CHEERS asks about fruit and vegetable provision, dark leafy greens, high fiber food, and fatty foods which are also captured in the EPAO-Foods Provided subdomain. Additionally, the document review of the nutrition policy in the EPAO includes an evaluation section that assesses the menu and foods and beverages served at the daycare center corresponding closely with the constructs measured in the Food Served subscale of CHEERS. The alignment between the self-report data and observational measurements provides further evidence of concurrent validity for the CHEERS audit tool.

We anticipated significant correlations between the Healthy Eating Environment Subscale of CHEERS and the EPAO-Feeding Environment and Feeding Practices, as they measure similar aspects such as “*using food as reward/bribe*,” “*encouraging children to try new foods*,” “*engaging in pleasant conversations during meal time*,” “*allowing children to decide what and how much to eat.*” However, no significant associations were observed. The lack of correlation could be attributed to various factors. One explanation may be that CHEERS serves as an audit tool for educators to self-assess their practices and create action plans accordingly. As in all self-assessment tools, educators may have a tendency to overreport favorable behaviors (64). It is also plausible that educators initially perceive their feeding practices as optimal based on their subjective evaluation. However, as they engage in the intervention and complete the different trainings to gain a deeper understanding of what really constitutes optimal nutrition practices and recommended guidelines, it is possible that educators may reassess and evaluate their practices differently by the end of the intervention. Overall, the observed discrepancies highlight the need for the intervention to address best nutrition practices and to examine changes in the scores of both assessment tools before and after the intervention.

### 4.1 Strengths and limitations

The quasi-experimental design of our study can be viewed as a limitation. However, including centers based on their willingness and capacity to implement the intervention provides a more realistic insight into how such programs might be embraced in real-world day care settings. Additionally, no significant baseline differences were observed between intervention and control group, except for age, which is controlled for in all analyses. Another limitation of this study is that most data were self-reported and the possibility of educators reporting a more favorable picture of their practices cannot be ruled out. However, to balance this we also used an objective observational measure (EPAO). Furthermore, although ECEC centers were randomly selected, the study’s scope was limited to ECEC centers in the province of Alberta which limits the generalizability of the findings. Additionally, the majority of participants (94.6%) were women which brings into question the representativeness of the study sample. However, this aligns with the gender distribution in the ECEC workforce as per the latest statistics (96%) (65).

One of the strengths of this intervention is the use of both quantitative and qualitative research methods for the implementation of the intervention and analysis of results. This triangulation of data will contribute to a more comprehensive evaluation of the intervention, its impact and future maintenance.

## 5 Conclusion

To our knowledge the CHEERS program is the first eHealth intervention tool in Canada that comprehensively addresses both the nutrition and physical activity social and physical environment in ECECs. The development of the CHEERS program was based on evidence and theory-driven methodology and shows great potential of being a practical and sustainable scaling up approach to promoting healthier early childhood education and care settings.

## Data availability statement

The datasets presented in this article are not readily available because the datasets generated during and/or analyzed during the current study are not publicly available due to participants’ privacy under the REB approval. Requests to access the datasets should be directed to LL, llafave@mtroyal.ca.

## Ethics statement

The studies involving humans were approved by the Human Research Ethics Board at Mount Royal University (no. 101768). The studies were conducted in accordance with the local legislation and institutional requirements. The participants provided their written informed consent to participate in this study.

## Author contributions

LL: Conceptualization, Data curation, Formal Analysis, Funding acquisition, Investigation, Methodology, Project administration, Resources, Supervision, Validation, Visualization, Writing – original draft, Writing – review and editing. JH: Data curation, Formal Analysis, Investigation, Methodology, Supervision, Validation, Writing – original draft, Writing – review and editing. AW: Conceptualization, Data curation, Investigation, Methodology, Resources, Validation, Writing – review and editing. CM: Conceptualization, Data curation, Investigation, Methodology, Resources, Validation, Writing – review and editing.
