# Supplementary material for: Creating healthy eating and active environments in early learning settings: protocol of the CHEERS eHealth intervention study
Source: Front Nutr. 2024 Feb 28;11:1337873. doi: 10.3389/fnut.2024.1337873 (PMC10932976; doi:10.3389/fnut.2024.1337873)
Supplement: Supplementary file 1 [file Data_Sheet_1.PDF]

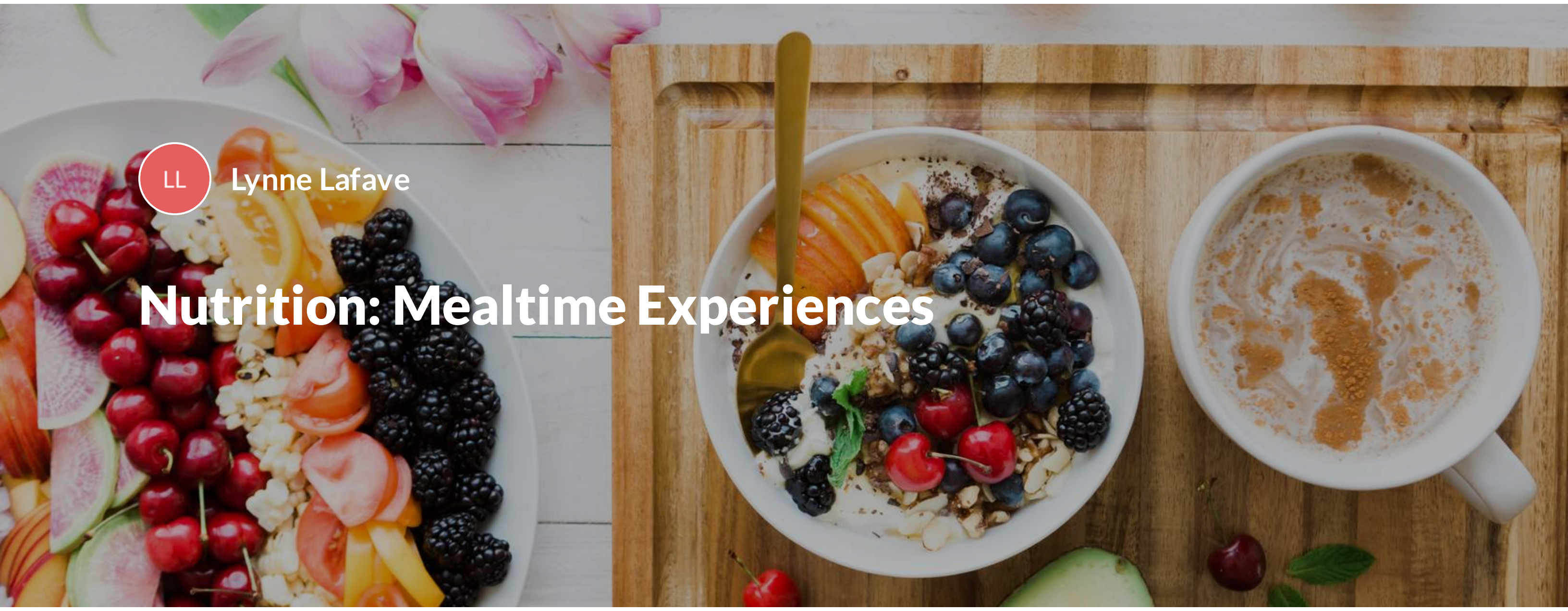

Lynne Lafave

Nutrition: Mealtime Experiences

CREATING HEALTHY EATING AND ACTIVE ENVIRONMENTS
CHEERS
IN EARLY LEARNING

The eating habits you teach a child in the early years can form a pattern that lasts a lifetime. Mealtimes are an opportunity for children to learn and practice eating, language and social skills. In this module, you will learn about how to create a positive mealtime experience.

In this module you will learn:

1. The reasons why a positive mealtime experience is important.
2. To identify strategies for creating a positive mealtime experience.
3. Why it is important to sit with children during mealtime.
4. Ideas for conversation starters at mealtime.
5. Why a healthy eating policy is important and how it can benefit your program.

Create a Positive Mealtime Experience

Lead by Example

Conversations at Mealtimes

Healthy Eating Policy

AUTHORSHIP

References

Create a Positive Mealtime Experience

Welcome to Module 7! We are so excited to have you learn with us.

Introduction

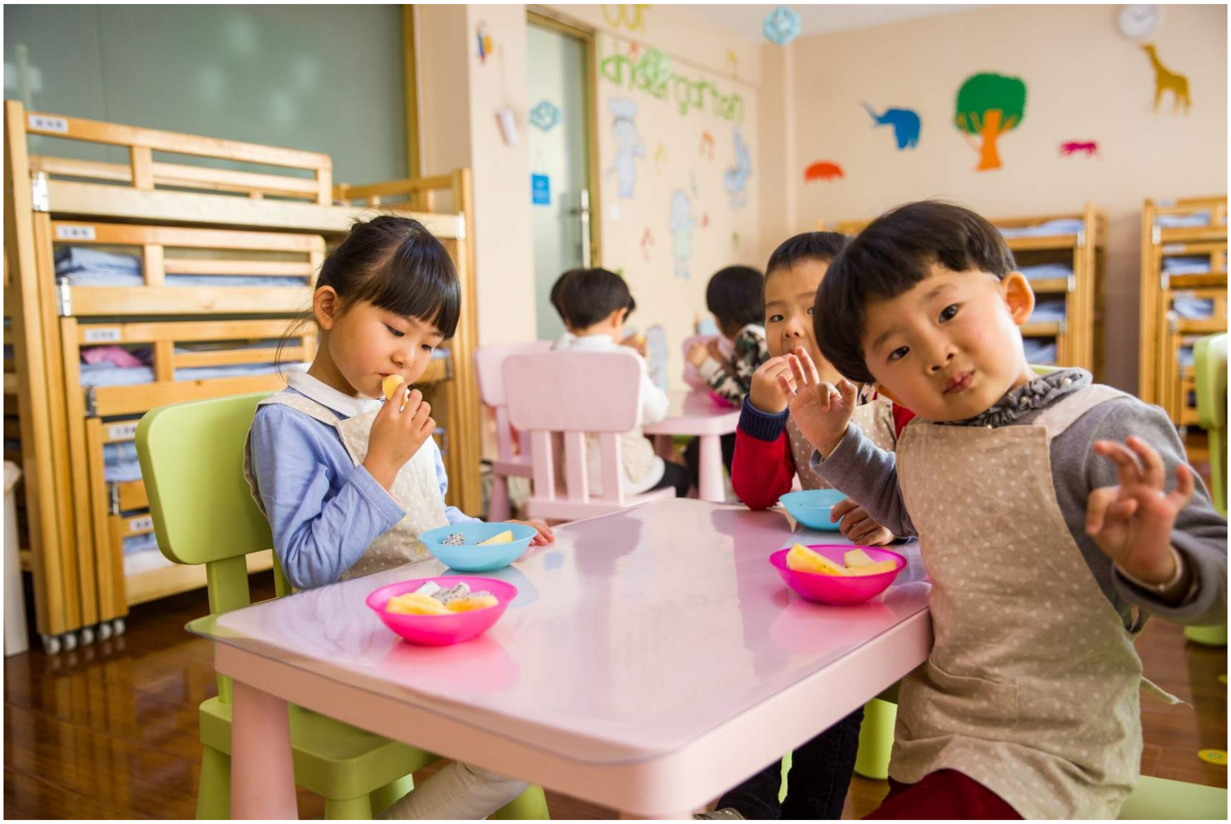

Create a Positive Mealtime Experience

Support positive mealtimes by establishing routines and transitions. Create a child-friendly space to help children feel settled and comfortable at mealtime. Learn about a number of ways your program can provide a positive mealtime experience for children.

Learning Objectives

For module 7.1

1. Explain 2 reasons why a positive mealtime experience is important.
2. Identify 3 strategies for creating a positive mealtime experience.

CONTINUE

What is a Positive Mealtime Experience?

A positive mealtime experience is planned and purposeful to support a child's desire to learn. It includes the social and physical environments, and the foods offered. A positive mealtime experience promotes a child's social connections, learning, and health during meal and snack times.

Click on each of the plus signs (+) on the image below to reveal more information.

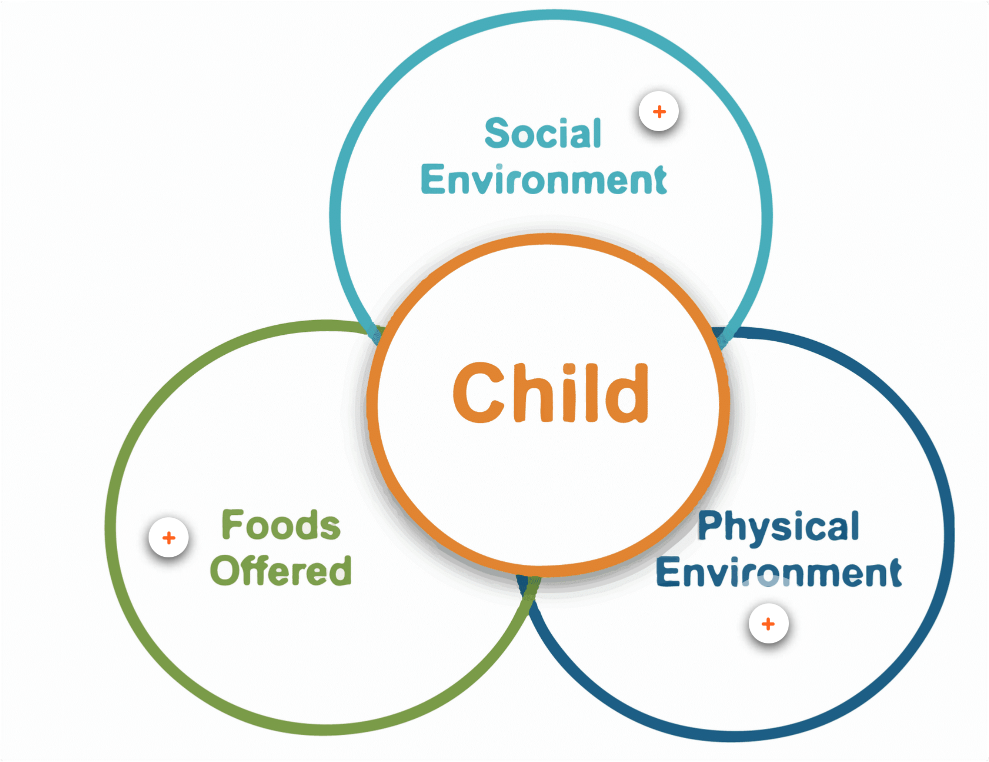

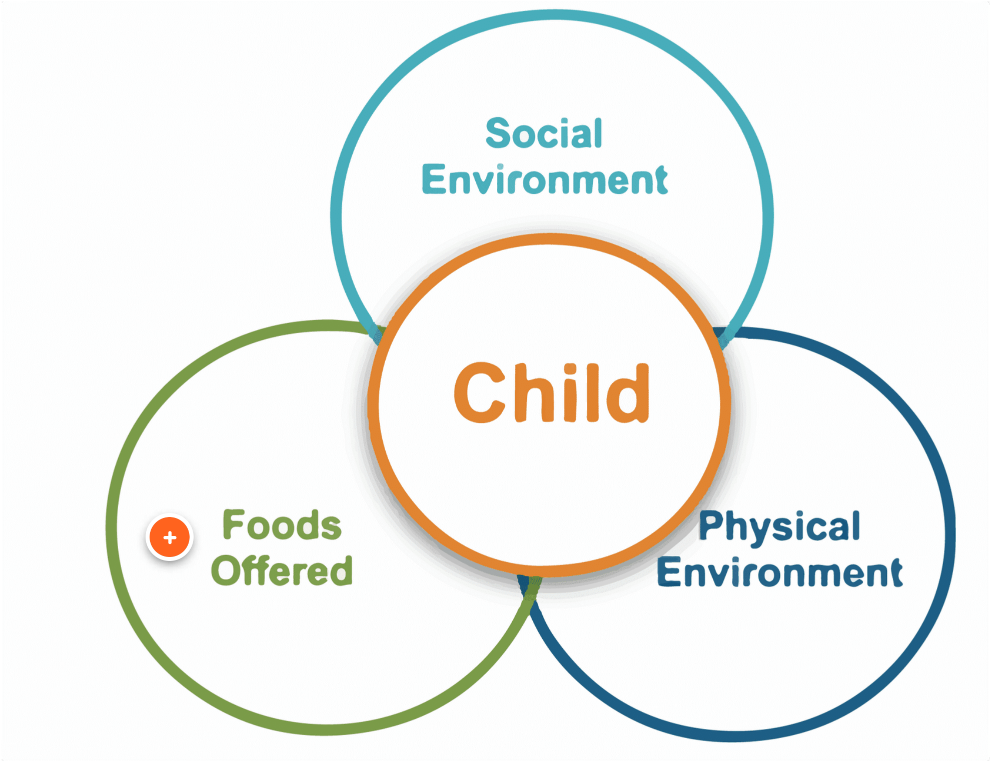

Food Offered

Offer a variety of healthy food from Canada's Food Guide ↗ including cultural and traditional foods.

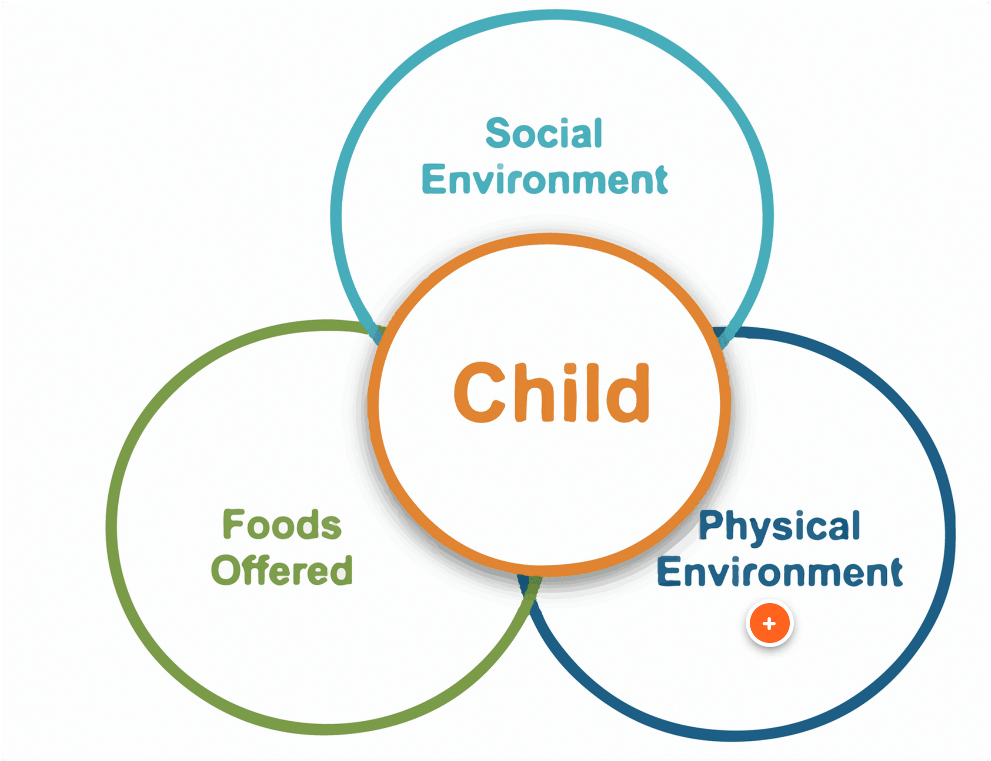

Physical Environment

Create a child friendly space with age-appropriate equipment such as tables, chairs and dishes. Support positive mealtimes by establishing routines and transitions, and avoiding distractions.

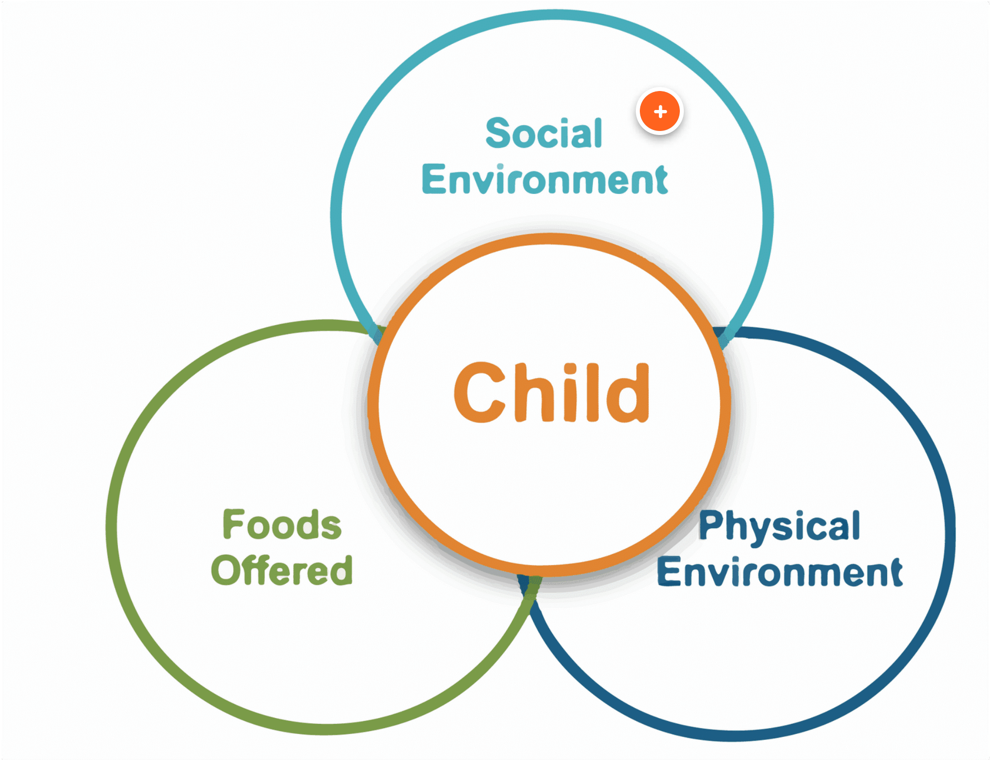

Social Environment

Make mealtimes pleasant and relaxed. Enjoy sitting, talking, and eating together. Encourage learning by involving children, following the feeding relationship, and offering family-style meals.

CONTINUE

Why is a Positive Mealtime Experience important?

The mealtime habits you teach a child in the early years can form a pattern that lasts a lifetime.

It is important to create a positive mealtime environment for children to:

- Promote a relaxed and pleasant mealtime environment.
- Strengthen relationships while sharing meals.
- Help children learn about food and their eating preferences.
- Help children meet their energy and nutrition needs to support healthy growth and development.
- Allow children to follow their hunger and fullness cues.

(Source: Alberta Health Services, 2020; Ellyn Satter Institute)

Structure, Routine and Transitions

Knowing that meals and snacks are at regular times helps children feel safe about eating. They are less likely to worry when their next meal or snack will be served.

Tips

- Plan meals and snacks 2 to 3 hours apart. This helps children feel ready to eat at mealtime.
- Treat snacks as "mini-meals" that still consider children's nutritional requirements.

- Allow about 20 to 30 minutes to eat meals and 10 to 15 minutes to eat snacks.
- If the food is not eaten within this time, let the child leave the table.
- Invite children to the table to visit and socialize, even if the child does not want to eat.

A transition before meals and snacks may help your child prepare for eating.

Here are some examples that you may find helpful:

- 5-minute advance notice
- Pick up toys
- Wash hands
- Help set the table

(Source: Alberta Health Services, 2018; Alberta Health Services, 2016, Ellyn Satter Institute)

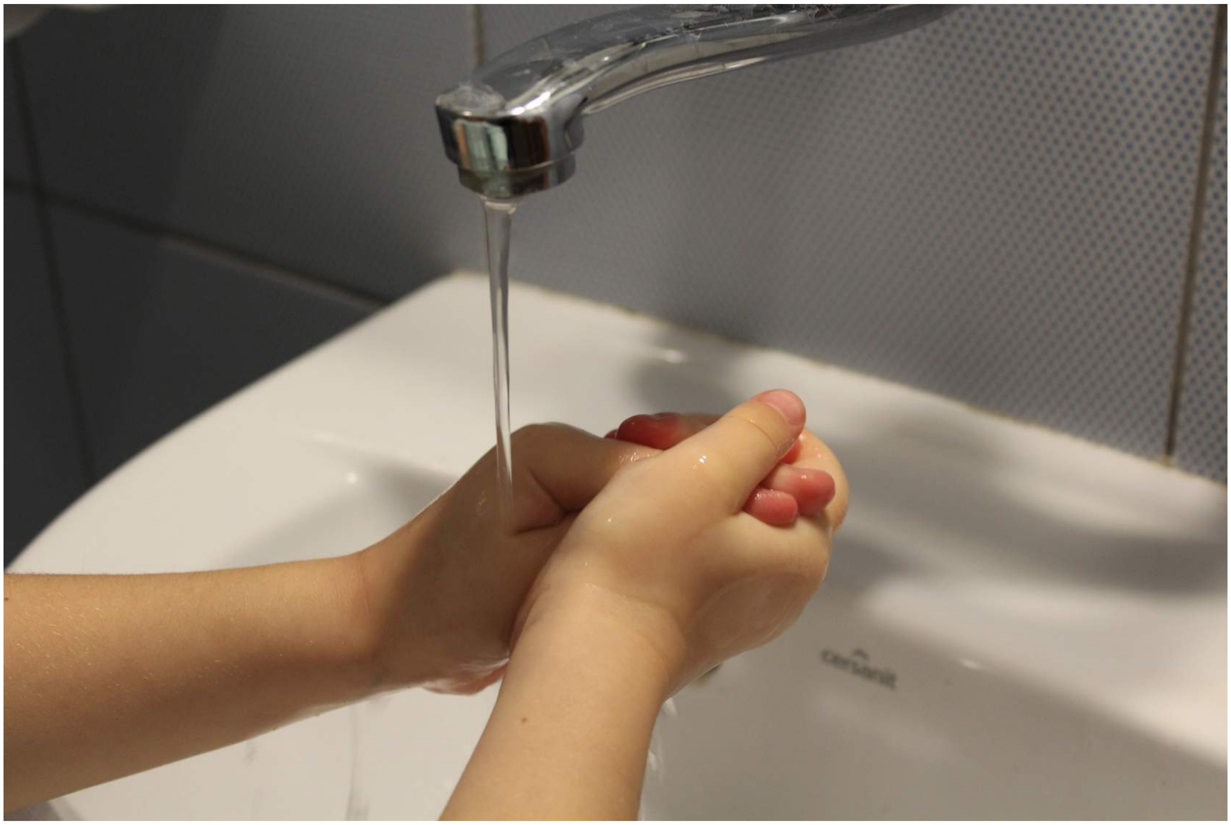

CONTINUE

Create a Child-Friendly Space

Try it out! Sit on the edge of your chair, lift your feet off the floor and pretend to eat a meal. How does this feel? Probably not very comfortable.

It is important for children to feel supported while eating:

SITTING	DISHES	FAMILY-STYLE

Children eat best when they are sitting comfortably, with their back and feet supported.

(Image Source: iStock.com/Orbon Alija)

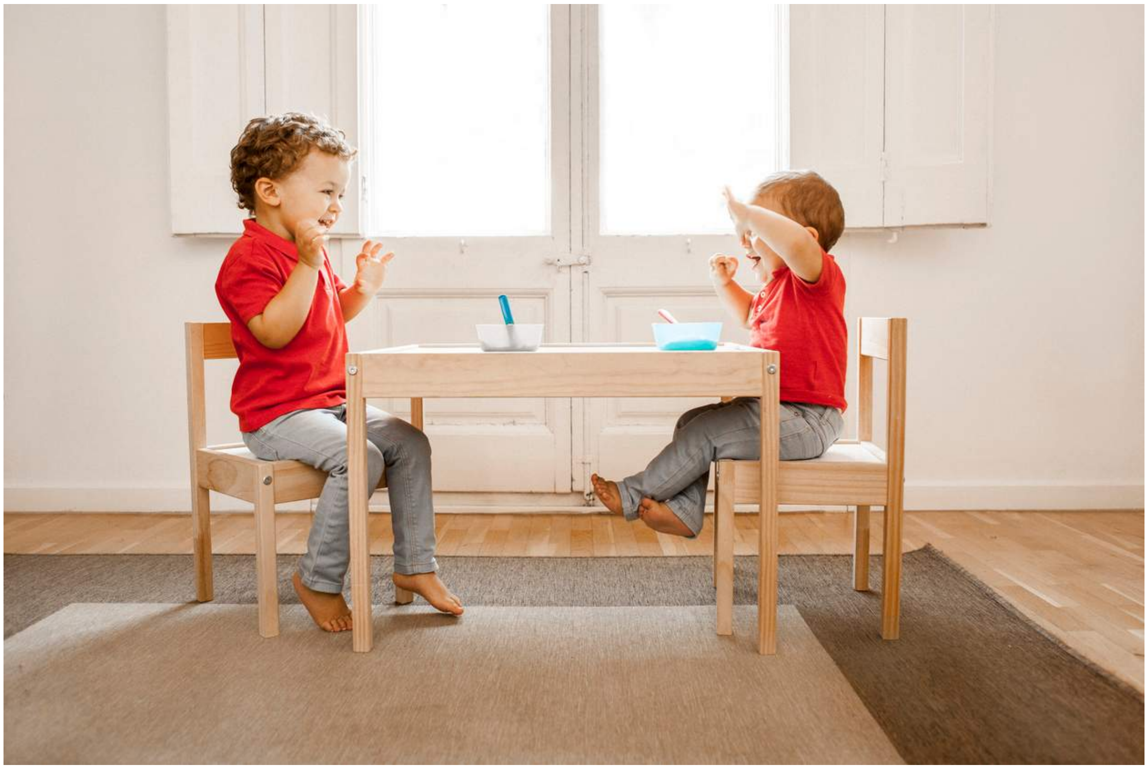

SITTING | DISHES | FAMILY-STYLE

Use child-sized serving utensils, dishes, and cups.

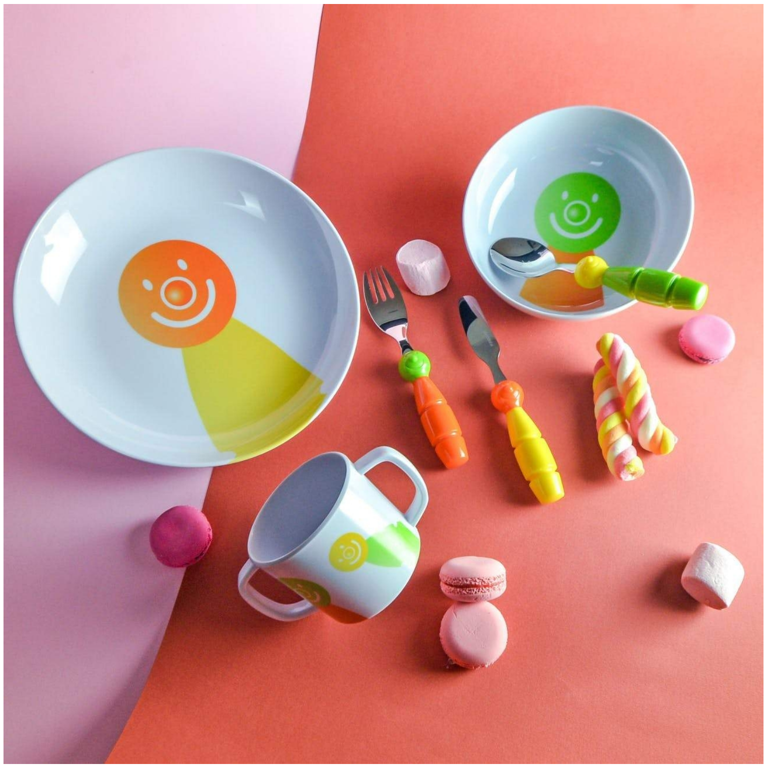

SITTING | DISHES | FAMILY-STYLE

Learn more about family-style meal service ↗

(Image Source: iStock.com/vaaseenaa)

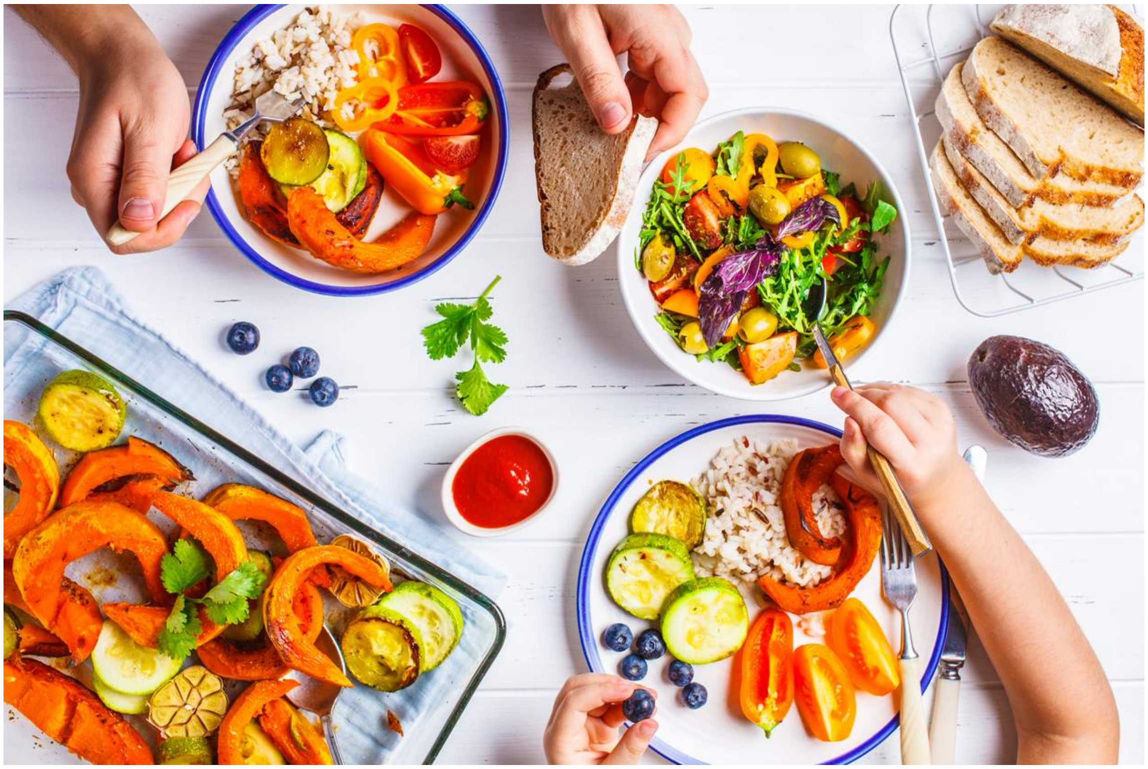

Creating a child-friendly space helps children feel settled and comfortable. This allows children to focus on eating as well as listening to their internal hunger and fullness cues.

(Source: Alberta Health Services, 2018)

CONTINUE

Knowledge Check!

Which of these strategies would help create a positive mealtime experience?

- ◯ Planning regular meals and snacks every 2-3 hours.
- ◯ Offering children small sized cups for drinking beverages.
- ◯ Talking to children about their favourite animal during mealtime.
- ◯ All of the above

SUBMIT

CONTINUE

Reflection Time

Let's take a moment to reflect.

Use the box below to answer the following questions. Remember to click "Done" to submit your answer.

Nutrition Reflection

* 1. Give an example of how you can provide a positive mealtime experience in your setting?

Lead by Example

Introduction

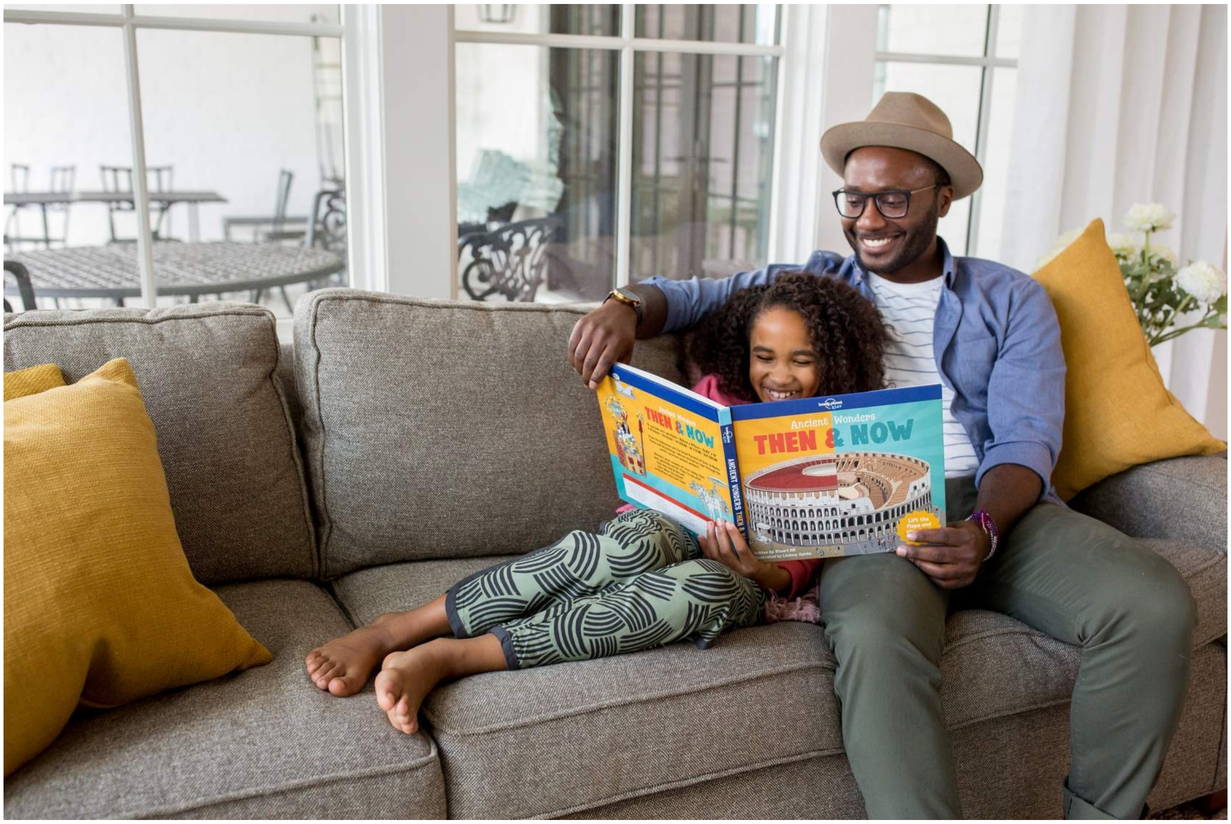

Lead by Example

Eating food gives children the energy and nutrition needed to grow, learn, and play. Children learn about food and eating by watching others, especially by

watching their caregivers. Be a positive role model. Remember that the eating habits and attitudes children develop now are going to affect their habits and attitudes later on in life.

Learning Objectives

For module 7.2

1. Explain why it is important for Early Childhood Educators to sit with children during mealtime.

CONTINUE

Sit and Eat with Children During Meal and Snack Times

Children learn to eat by watching you!

Be a positive role model by sitting with children. Let them see you eating and enjoying a variety of healthy food. This will help children learn to eat well and explore new foods.

(Source: Alberta Health Services)

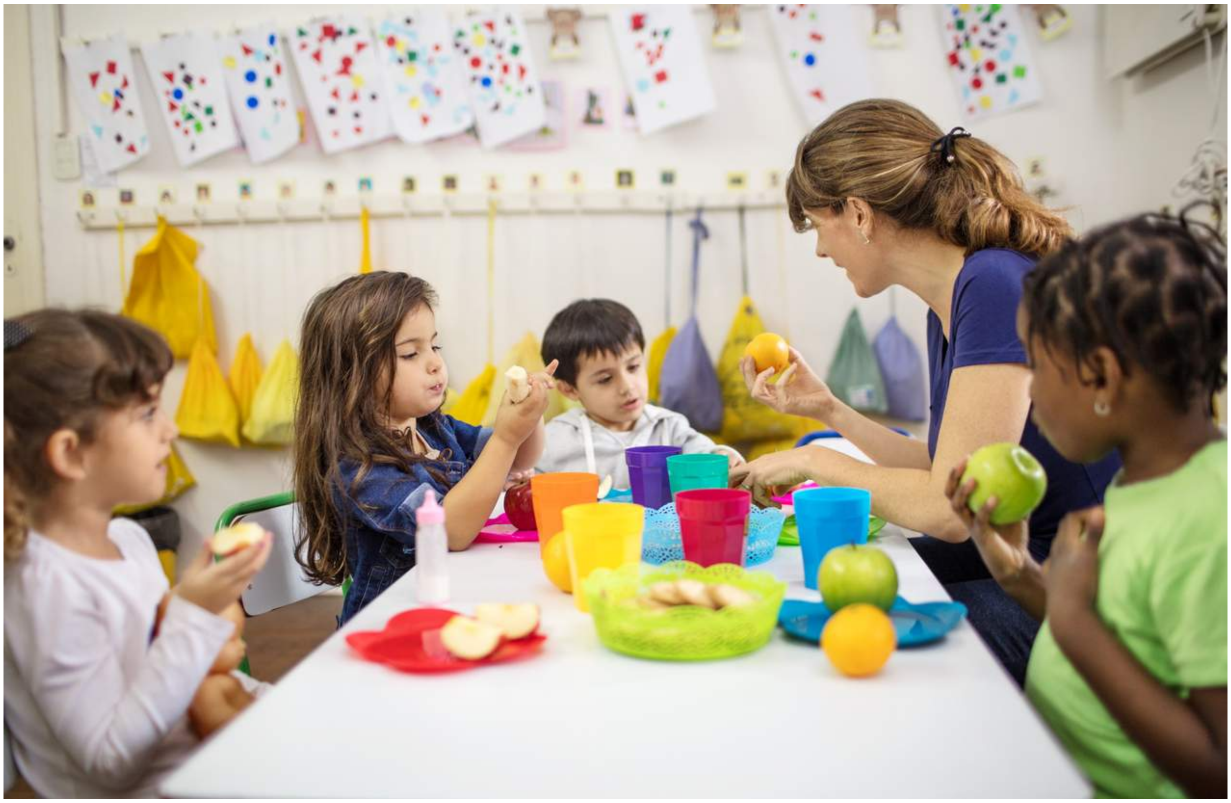

(Image Source: iStock.com/alvarez)

Children learn and practice new skills at mealtime by watching you:

- Table manners
- Feeding skills such as eating with a fork and spoon
- Passing/serving food
- New vocabulary and language skills
- Exploring colours, shapes, temperature, counting, etc.

See Alberta Health Services poster, Eat Together ↗

CONTINUE

Avoid Distractions

Remove distractions to help children focus on eating and listening to their internal fullness and hunger cues.

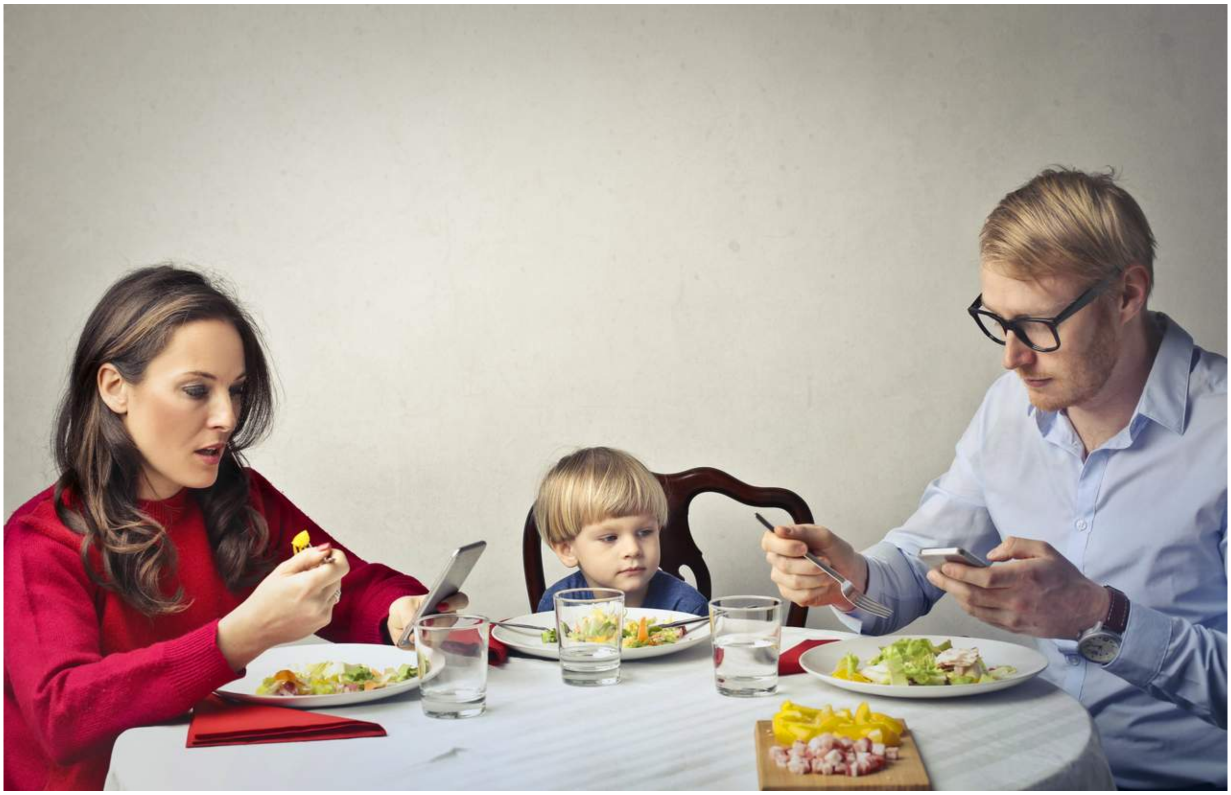

Example of Negative Role Modelling

(Image Source: iStock.com/bowie15)

Tips

- Turn off the TV
- Put aside toys, phones, and electronics while eating
- Talk together
- Keep mealtimes pleasant and relaxed
- Focus on each other and the meal

CONTINUE

Eat Together

This video discusses the benefits of enjoying meals with children.

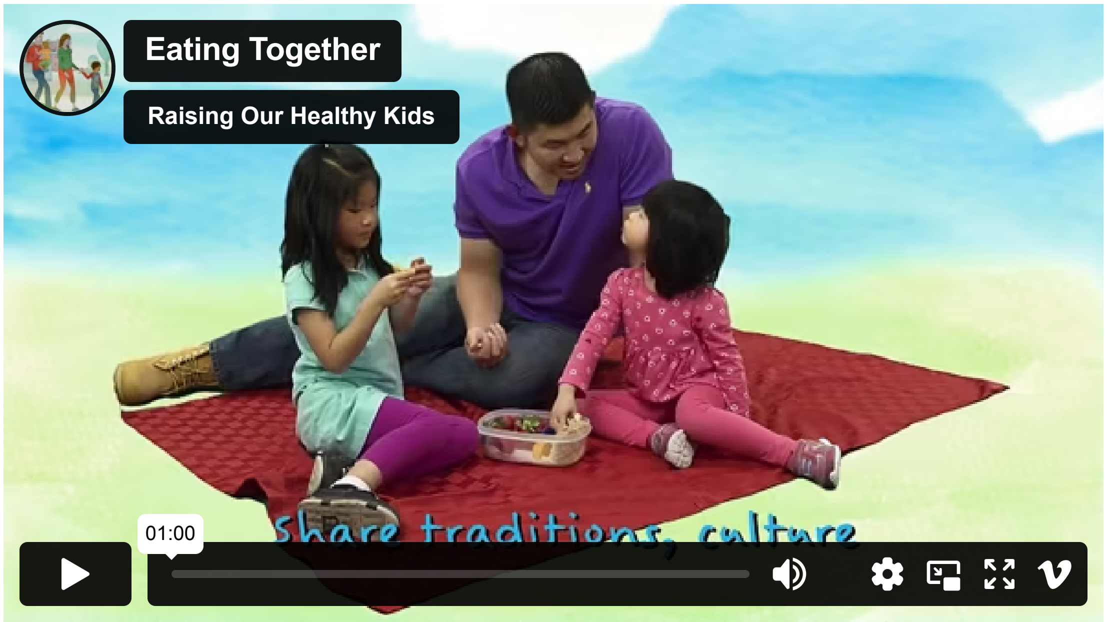

Mealtimes are a great time for you and the children to visit and talk. Keep mealtimes pleasant and relaxed. Let children see you enjoying a variety of foods. This will help children try new foods and to learn eating skills.

CONTINUE

Role Model Healthy Eating

This video reviews the importance of role modelling healthy eating for children.

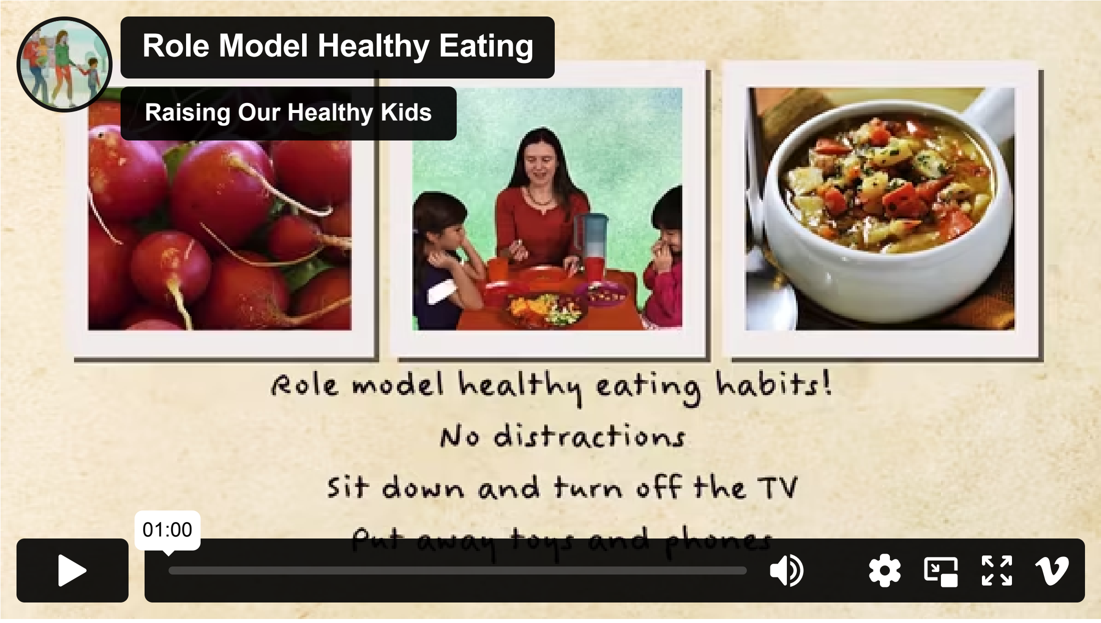

Children learn healthy eating habits by watching their caregivers. Role model positive attitudes toward healthy food. When you eat healthy foods, children will be more likely to do the same.

CONTINUE

Knowledge Check!

Drag and drop the item to the category it belongs.

Positive Role Modelling

Sitting with children

Showing how to eat yogurt with a spoon

Talking about the shape of broccoli

Negative Role Modelling

Answering a text from a friend

Commenting how much you dislike zucchini

Watching a video on your iPad

CONTINUE

Reflection Time

Let's take a moment to reflect.

Use the box below to answer the following questions. Remember to click "Done" to submit your answer.

Nutrition Reflection

* 1. Why is it important to sit with children at mealtime?

Conversations at Mealtimes

Introduction

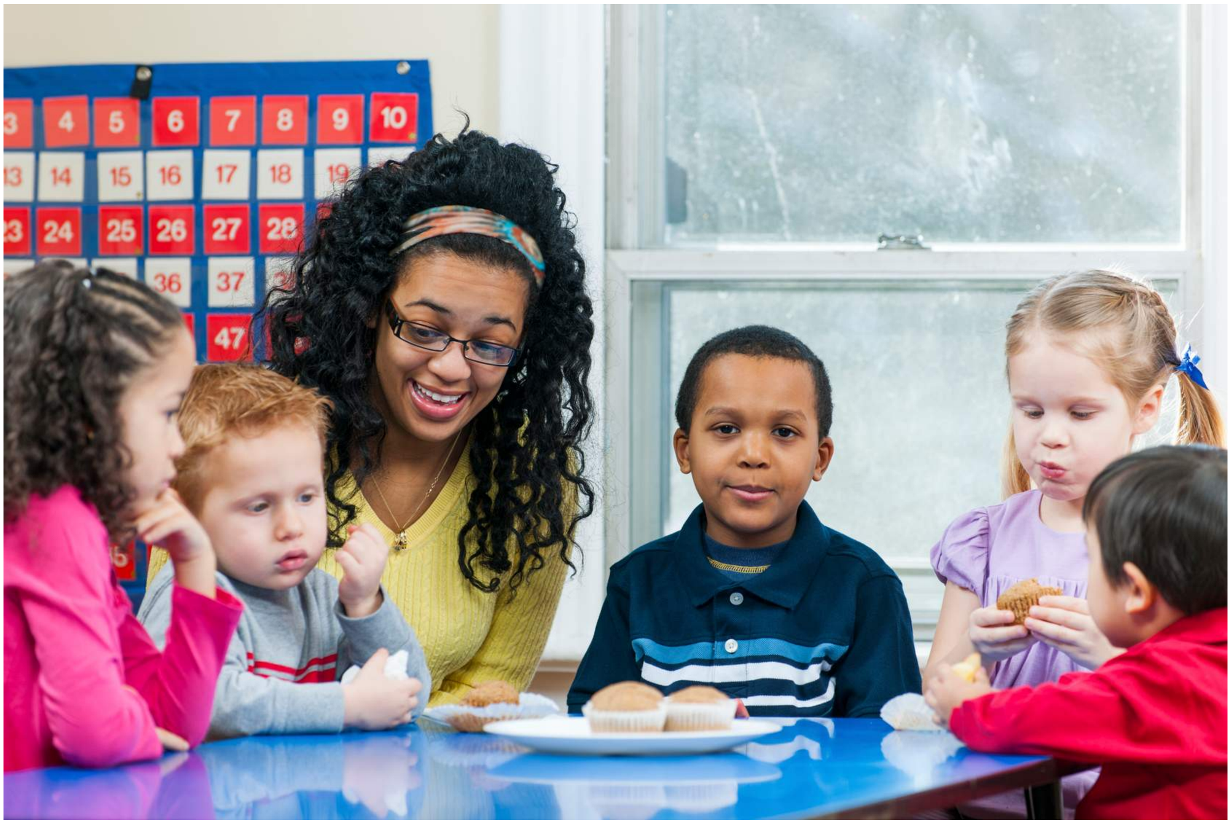

Engage in Conversation at Mealtime

Mealtimes are an opportunity for children to learn and practice language and social skills.

Learning Objectives

For module 7.3

1. Name 2 ideas for conversation starters at mealtime.

CONTINUE

Talk Together

Positive conversation:

- Creates relaxed meal and snack times.
- Builds relationships.
- Allow children to practice language and social skills, and learn about food and eating.

(Source: Alberta Health Services, 2018)

Conversation Starters

Keep the conversation positive to make meal and snack times enjoyable. Involve children in the conversation. Ask short questions and questions that need more than a one-word answer.

(Source: Alberta Health Services, 2018)

Ideas to spark conversation:

- What was the best part of your day?
- What is your favourite animal? Why?
- What does a ______ taste like?
- What shapes/colours are on your plate?
- What celebration do you enjoy the most?
- What is the best thing about being ___ years old?

(Source: Alberta Health Services, 2018)

For more conversation ideas, see Alberta Health Services' Conversation Cards ↗

CONTINUE

Knowledge Check!

True or False: Children learn by talking with caregivers at mealtime.

True

False

SUBMIT

CONTINUE

Reflection Time

Let's take a moment to reflect.

Use the box below to answer the following questions. Remember to click "Done" to submit your answer.

Nutrition Reflection

* 1. Name two conversation starters you can use at mealtime.

Healthy Eating Policy

Introduction

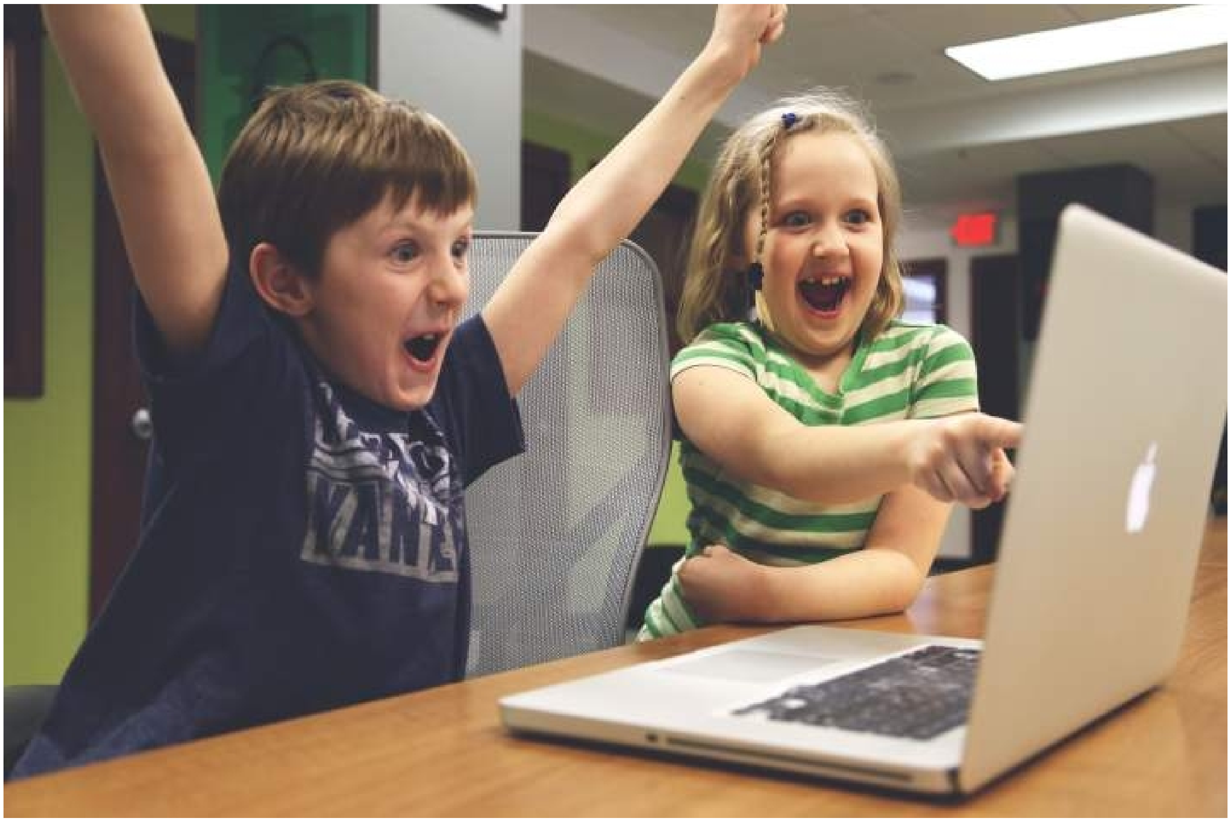

Healthy Eating Policy

A written healthy eating policy supports educators, families and children.

When developing a healthy eating policy, it is important to consider: offering

healthy foods, creating a healthy eating environment, and integrating nutrition education in the curriculum. Communicate with families about healthy eating activities in your program.

Learning Objectives

For module 7.4

1. Explain why a healthy eating policy is important and how it can benefit your program.

CONTINUE

Why do child care programs need healthy eating policies?

What is a Policy?

A policy is a clear written statement that people are required to follow. It provides a set of guidelines to guide practice and help make decisions.

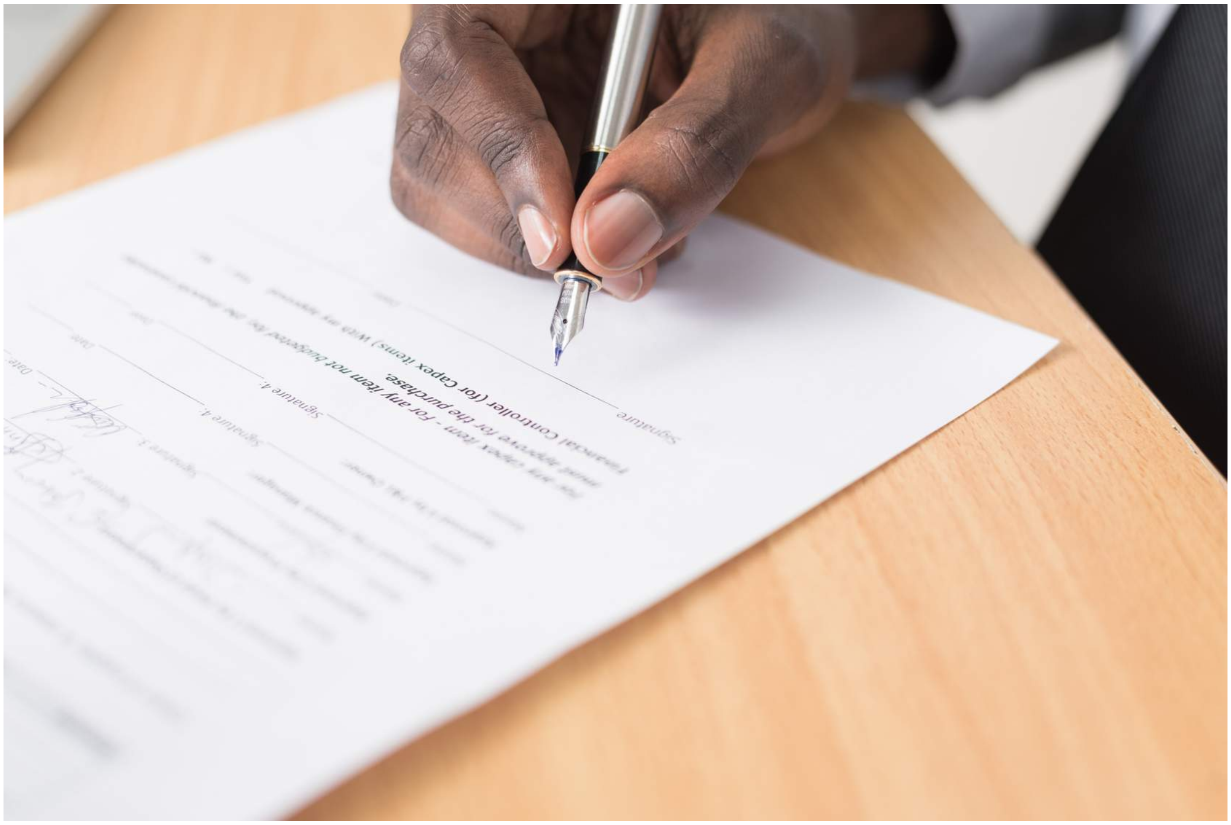

Why is a Policy Important?

- It supports children's learning, development, and health.
- It helps guide educators make choices about the best practices to follow.

Why do child care programs need healthy eating policies?

Healthy eating policies can help:

- Create a plan for providing food and beverages that are healthy, safe, and varied.

- Comply with government regulations.
- Set expectations and roles for educators and parents.
- Give clear steps for managing special needs (i.e. allergies) and special occasions (i.e. birthdays).

(Source: OPDH)

CONTINUE

Thinking about Developing a Nutrition Policy

When developing a healthy eating policy for a child care program, it is important to consider the following areas:

1

Offer Healthy Foods

Example:

Our program offers a variety of healthy foods and drinks from Canada's Food Guide ↗:

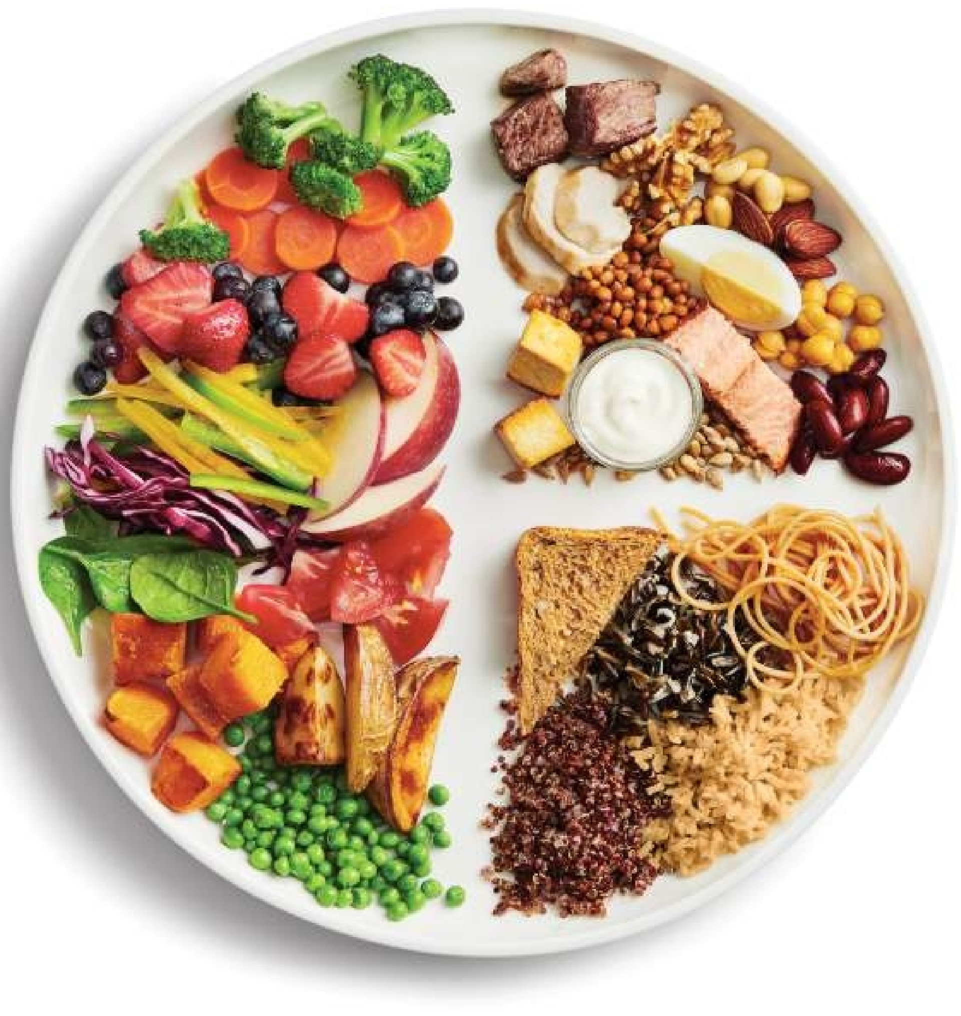

1. Meals include vegetables and/or fruit, whole grains, iron-containing protein foods (i.e. chicken, beans, fish, meat).
2. Milk is offered at meals.
3. Snacks include vegetables or fruit, along with whole grain foods and/or protein food.
4. Water is available throughout the day.
5. Sugary drinks and highly processed foods (i.e. hot dogs, French fries, candy) are not offered to children.

If families provide food or beverages, they are encouraged to offer a variety of healthy foods from Canada's Food Guide.

2

Create a Healthy Eating Environment

Example:

Our program creates a positive mealtime experience by offering regular meals and snacks. We allow the child to decide how much to eat and whether to eat from the foods offered.

Educators lead by example by:

1. Promoting a relaxed atmosphere.
2. Sitting and talking with children.
3. Teaching table manners, self-help, and language skills.

3

Healthy Eating Education & Programming

Integrate nutrition education or programming into activities and learning opportunities throughout the day.

Example:
We integrate nutrition education into our daily programming to help children explore and learn about healthy food choices.

You will learn more about this in the next nutrition module!

CONTINUE

Healthy Eating Policy Example

[Date policy was developed]
[Child care program] creates a healthy eating environment by role modelling healthy eating behaviours and allowing children to decide how much to eat from what is offered.

At Meals and Snacks:

- Educators follow the division of responsibility in regards to feeding children. Children decide how much to eat from the foods offered at meals and snacks, and whether to eat.
- Educators do not use food bribe, reward, or pressure children.

- At least one educator sits with the children during meal and snack times.
- Educators encourage mealtimes that are pleasant with positive conversations.
- Children are seated at a table or in a high chair when eating.
- There are no toys, books, screens, or other distractions at the table while eating
- Children are given at least 20 minutes to eat meals and snacks
- Educators involve children in some aspect of mealtimes (i.e. setting or clearing table).

[Date approved]

Approved by:

(Adapted from ODPH)

CONTINUE

Inform Families

It is helpful to communicate with families about:

- The policies, procedures and activities that support healthy eating environments in your program.
- How their child is learning about and practicing healthy eating.

Examples:

1. **At registration**: review your healthy eating policies or provide a parent handbook containing these policies.
2. **Post** your healthy eating activities for families to see – include photos of it being implemented and tips for families.
3. **Post** photos of foods offered on that day's menu to increase familiarity with the menu.
4. **Post** a 'question of the week' to be used as conversation starters between program staff and families, as well as the parents and child.
5. **At pick up time**, share specific examples of healthy eating activities that their child participated in that day.

(Source: Alberta Health Services, 2017)

CONTINUE

Knowledge Check!

What are important areas an Early Learning and Child Care program should include in a healthy eating policy?

a. Offer processed food

b. Create a healthy eating environment

c. Integrate nutrition education into activities throughout the day

d. b and c

SUBMIT

CONTINUE

Reflection Time

Let's take a moment to reflect.

Use the box below to answer the following questions. Remember to click "Done" to submit your answer.

Nutrition Reflection

* 1. Do you have a healthy eating policy in your program? If yes, what does it say?

CONTINUE

Key Takeaways

- Support positive mealtimes by establishing routines and transitions, and by creating a child friendly space.
- Sit with children at meal and snack times to lead by example.
- Mealtimes are an opportunity for children to learn and practice language and social skills. Talk with children at meals and snacks.

- A written healthy eating policy supports staff and families in creating a healthy eating environment.

Congratulations, you've finished the module! Thank you for completing it, we hope you enjoyed learning with us.

References

Contributor

Kristen Di Lullo

BSc., RD

Kristen has over 6 years of experience working as a Registered Dietitian in a variety of public health and clinical settings. Through her work as a public health dietitian, she has a strong passion for health promotion, and has contributed too many health-focused initiatives to support communities and families in Alberta.

Special thanks to the registered dietitians on the Population and Public Health team at Alberta Health Services for their contributions to the nutrition content in these modules.

References

Alberta Health Services, Health Eating Starts Here – Early Learning & Child Care Staff Training: Eat Together! Poster, 2016.

Alberta Health Services, Healthy Eating Starts Here – Early Learning & Child Care Staff Training: Feeding Toddlers and Young Children, 2016.

Alberta Health Services, Healthy Eating Starts Here –Early Learning & Child Care Staff Training: Children learn to eat by watching you! Poster, 2016.

Alberta Health Services, Healthy Eating Starts Here: Early Learning & Child Care Staff Training: Talk Together Poster, 2018.

Alberta Health Services, Information for Your Business – Environment Public Health: Family-Style Meal Service in Child Care Programs, 2018.

Alberta Health Services, Mealtime Struggles Class Notes, 2018.

Alberta Health Services, Mealtime Struggles Class Notes, 2018.

Alberta Health Services, CHEERS Virtual Communities of Practice (VCoP) Grant Material – Session 7: Parents as Partners, 2017.

Alberta Health Services, Supporting Positive Mealtime Resources, 2020.

Ellyn Satter Institute. (n.d.). *Family meals and snacks*. https://www.ellynsatterinstitute.org/how-to-eat/family-meals-and-snacks/

ODPH, Paint Your Plate Toolkit: A Toolkit For Ontario Child Care Providers.

Ontario Dietitians in Public Health, Paint Your Plate Toolkit: A Toolkit For Ontario Child Care Providers, Date Accessed [29 Jul 2019].
